# High serum CCL18 predicts a poor prognosis in patients with laryngeal squamous cell carcinoma

**DOI:** 10.7150/jca.37515

**Published:** 2019-11-17

**Authors:** Juncheng Wang, Yuexiang Qin, Gangcai Zhu, Donghai Huang, Ming Wei, Guo Li, Li She, Diekuo Zhang, Gang Wang, Xiyu Chen, Zhe Shen, Yuanzheng Qiu, Yunyun Wang, Haolei Tan, Pingqing Tan, Jie Chen, Xin Zhang, Yong Liu

**Affiliations:** 1 Department of Otolaryngology Head and Neck Surgery, Xiangya Hospital, Central South University, 87 Xiangya Road, Changsha, Hunan 410008, People's Republic of China.; 2 Otolaryngology Major Disease Research Key Laboratory of Hunan Province, 87 Xiangya Road, Changsha, Hunan 410008, People's Republic of China.; 3 Department of Health Management, the Third Xiangya Hospital, Central South University, 138 Tongzipo Road, Changsha Hunan 410013, People's Republic of China.; 4 Department of Otolaryngology Head and Neck Surgery, the Second Xiangya Hospital, Central South University, 139 Renmin Road, Changsha, Hunan 410011, People's Republic of China.; 5 Department of Head and Neck Surgery, Hunan Cancer Hospital, The Affiliated Tumor Hospital of Xiangya Medical School, Central South University, 283 Tongzipo Road, Changsha, Hunan 410013, People's Republic of China.

**Keywords:** CCL18, LSCC, Prognosis, Tumor associated Macrophage.

## Abstract

CCL18 is a cytokine secreted by M2 type tumor associated macrophages, which frequently over-expressed in diverse human cancers. However, the clinical significance of serum CCL18 in patients with laryngeal squamous cell carcinoma (LSCC) remains unknown. In this study, serum CCL18 was initially quantified by enzyme-linked immunosorbent assay (ELISA) in 146 patients with LSCC, 25 patients with precancerous lesions and 72 healthy volunteers. In addition, the correlations between serum CCL18 and clinicopathological parameters were analyzed. Our data revealed that serum CCL18 was obviously increased in patients with LSCC. Moreover, serum CCL18 level was significantly associated with primary tumor site (Glottic vs Others), T classification (T1+T2 vs T3+T4), clinical stage (I+II vs III+IV) and lymph node metastasis (N0 vs N+). Survival analysis demonstrated that patients with high serum CCL18 displayed a shorter survival time than those in patients with low serum CCL18. Importantly, serum CCL18 level and clinical stage were independent prognostic factors in patients with LSCC. Taken together, serum CCL18 could be used as a promising biomarker in patients with LSCC.

## Introduction

Laryngeal squamous cell carcinoma (LSCC), as one of the head and neck squamous cell carcinoma (HNSCC), has the highest rate of voice-related disabilities and a poor mortality 1. Therapy for patients with LSCC including surgery, radiotherapy and chemotherapy, which are used either alone or in combination has been improved over the last few decades 2, 3. Unfortunately, the life quality and survival status for patients with LSCC are far from satisfactory 4, 5. Cancer initiation and progression are closely linked with the aberrant expression and function of endogenous oncogenes or suppressors in cancer cells themselves, the remodeling of tumor microenvironment (TME) and the mutual interaction between cancer cells and the components of TME 6.

Tumor associated macrophages (TAMs) is a critical component in TME, which originates from circulating blood monocyte and then differentiates into M1 or M2 type TAMs once entry into tumor 7. TAMs especially M2 type in primary tumor site, stimulates angiogenesis, enhances metastasis and also represses anti-tumor immune response, which indicates the protumoral role of TAMs in TME 8-10. Therapeutic success in targeting these protumoral roles in preclinical models and in early clinical trials suggests that TAMs are attractive targets as part of immunotherapy in cancer treatment 11.

Cytokines and chemokines secreted by TAMs participate in the malignant progression of cancers. Among these secretions, CCL18 is an important cytokine that derived from M2 type TAMs 12. CCL18 has been identified to function importantly in the invasion and metastasis of human cancers including head and neck cancer 13-16. High enriched CCL18 was tightly related to poor prognosis in multiple solid cancers, such as pancreatic ductal adenocarcinoma 17, 18, ovarian cancer 19, gallbladder carcinoma 20 and gastric cancer 21 etc. However, the clinical significance and especially prognostic value of serum CCL18 in patients with LSCC remains unclear.

In this study, we evaluated the levels of serum CCL18 by Enzyme-linked immunosorbent assay (ELISA) and investigated the association of CCL18 expression with the clinicopathological variables in 146 patients with LSCC. Our results reveal that serum CCL18 is increased in patients with LSCC, which is linked with diverse malignant phenotypes of LSCC including advanced clinical stages, lymph node metastasis etc. More importantly, examination of serum CCL18 provides useful prognostic information for patients with LSCC.

## Material and methods

### Subjects

Blood serum samples from 146 patients with LSCC, whose age was ranged from 31 to 80 years (mean 50.6 ± 12.0 years), were collected prior to treatment. As controls, blood samples from 25 patients with precancerous lesions and 72 healthy volunteers were also collected. All the 146 patients with LSCC received standard treatment between September 2012 and April 2014 in the Department of Otolaryngology head and neck surgery at Xiangya Hospital, Central South University, Changsha, China. Patients enrolled in our study had to follow these inclusion criteria: Primary squamous cell carcinoma without other malignancies; no history of previous radiotherapy and chemotherapy. As showed in **Table 1,** the clinical data including age, gender, primary tumor site, T classification, clinical stage, histological grade and lymph node metastasis were recorded in detail. No other cancer history was recorded for them. Pathological tumor-node-metastasis (TNM) stage was determined according to the 7th American Joint Committee on Cancer staging system. The protocol was approved by the Medical Research Ethics Committee of Xiangya Hospital, Central South University, Hunan, China.

### Enzyme-linked immunosorbent assay (ELISA) for serum CCL18

All blood samples were collected before initial treatment. To avoid the effects of diet on the level of serum CCL18, all participants were told to fast more than 8 hours, and their blood samples were collected in the morning between 7 a.m. and 10 a.m. by venipuncture (BD Vacutainer Plus). Blood samples were allowed to clot at room temperature followed by centrifuging at 3000 rpm for 15 min, and then tested within 2 hours. Samples with hemolysis were excluded from the study. Serum CCL18 was determined by an ELISA commercial kit (R&D Systems, MN, USA) according to the manufacturer's instructions. Each experiment was performed in triplicate.

### Data collection and follow up

According to the contact information provided by patients, the following methods were used for following up: outpatient consultation, telephone and letter visits. Survival time of patients with LSCC was calculated from the date of surgery. Patient death, loss of follow-up or the last follow-up time were taken as the end point of the follow-up. 133 patients (91.1%) with LSCC accepted postoperational following up and detailed information was recorded, 13 patients failed to follow up due to changes in phone numbers and/or home address.

### Statistical analysis

The statistical analysis was performed using SPSS (version 22.0, SPSS Inc., Chicago, IL, USA). The Mann-Whitney U-test was used to analyze the differences between groups. A *P* value of <0.05 was considered as statistical significance.

## Results

### Serum CCL18 levels in patients with LSCC and healthy controls

**Figure 1:**
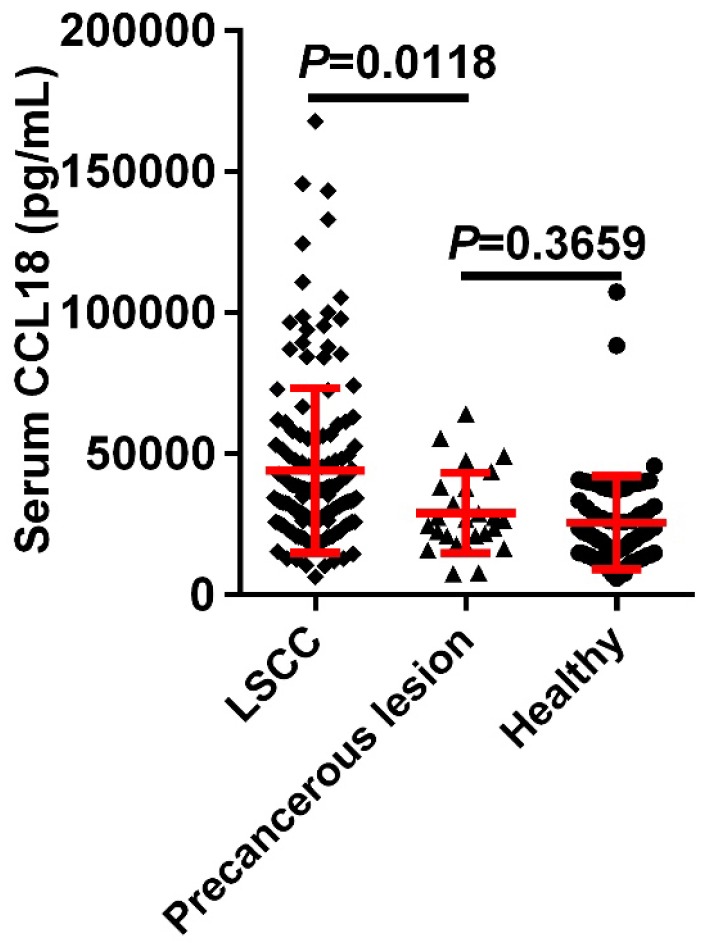
Serum CCL18 in patients with LSCC, patients with precancerous lesions and healthy volunteers was quantified by ELISA. Each dot represents one sample in each group.

To determine whether serum CCL18 were relevant to LSCC, peripheral blood samples were obtained from 146 patients with LSCC, 25 patients with precancerous lesions and 72 healthy controls. Our data showed that serum content value of CCL18 in patients with LSCC was 44086 ± 2408 pg/mL, while in those healthy individuals was 25588 ± 1938 pg/mL and in patients with precancerous lesions was 28945 ± 2851 pg/mL. The mean concentration of serum CCL18 in patients with LSCC was dramatically higher than that in patients with precancerous lesions and healthy individuals (**Fig.1**; *P* = 0.0118), which no difference existed between patients with precancerous lesions and healthy volunteers (*P* = 0.3659).

### Clinical associations of serum CCL18 in patients with LSCC

Since serum CCL18 was elevated in patients with LSCC, we further asked whether serum CCL18 levels correlated with the clinicopathological parameters in patients with LSCC. As illustrated in **Table 1**, serum CCL18 level was positively associated with primary tumor site (Glottic vs. Others; *P* < 0.001), tumor classification (T1+T2 vs. T3+T4; *P* < 0.001), clinical stage (I+II vs. III+IV; *P* < 0.001) and lymph node metastasis (N0 vs. N+; *P* < 0.001). However, there was no relationship between serum CCL18 and other parameters including age (*P* = 0.425), gender (*P* = 0.116) and histological grade (*P* = 0.404). Taken together, these data suggests that serum CCL18 level is a potentially valuable biomarker in the progression of LSCC.

### High serum CCL18 predicts a worse prognosis in patients with LSCC

**Figure 2:**
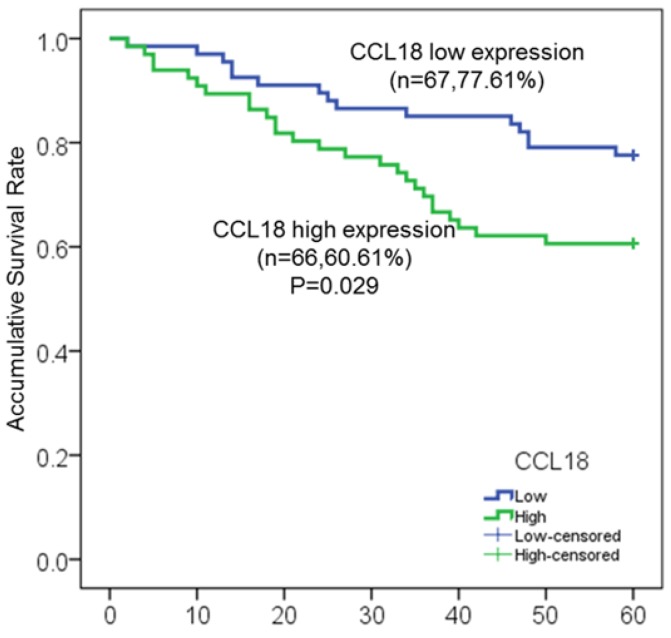
Kaplan-Meier survival curves of serum CCL18 high expression and low expression in patients with LSCC.

In our study, a total of 146 patients with LSCC were enrolled, in which 133 patients (91.1%) with intact clinicopathological information were used for further survival analysis. Among these 133 patients, 41 patients (30.8%) died at different time after surgery, and 54 patients (40.6%) experienced local recurrence by fibrolaryngoscopy, CT and MRI. To investigate the potential association between serum CCL18 level and prognosis, patients were divided into two groups including high serum CCL18 (n = 66) and low serum CCL18 (n = 67), in which serum CCL18 level was greater than or lower than the median value in our patient cohort. Therefore, Kaplan-meier method and log-rank test were then used to calculate the 5-year overall survival rate (60.61% vs. 77.61%; *P* = 0.029; **Fig.2**) in these two groups. Patients with high serum CCL18 had a shorter overall survival time (median survival time 40.8±9.7 months vs. 53.9±5.5 months). Collectively, these data reveals that serum CCL18 could be used as a predictor for the prognosis of patients with LSCC.

### Serum CCL18 level was an independent factor in patients with LSCC

Eight clinical parameters including age, gender, primary tumor site, histopathological grade, T staging, presence or absence of lymph node metastasis, clinical staging and serum CCL18 level were initially enrolled in univariate analysis. Univariate Cox regression analyses initially indicated that primary tumor site, T staging, clinical stage, lymph node metastasis and serum CCL18 level were significantly associated with the overall survival in patients with LSCC (All *P* < 0.05; **Table 2**). However, only clinical stage (*P* = 0.006; **Table 2**) and serum CCL18 level (*P* = 0.001; **Table 2**) were independent factors affecting the prognosis in patients with LSCC. The relative risk (RR) of CCL18 level in serum on the overall survival rate was 1.538, with a 95% confidence interval of [0.926-3.017].

## Discussion

TME is largely orchestrated by inflammatory cells, is an indispensable participant in the neoplastic process, which favors malignant cell proliferation, survival, metastasis and resistance to cancer therapy etc 22. M2-type TAMs, as one of the most critical components in TME, play a key role in the crosstalk between cancer cells and their surrounding TME 8. M2 type TAMs actively participate in diverse malignant processes such as tumor extracellular matrix remodeling, immune suppression and angiogenesis, which are accelerators that promotes cancer metastasis and resistance to therapy 9, 11, 23.

In our study, we found that CCL18, as a representative C-C cytokine, was overexpressed in the serum of patients with LSCC. Clinical significance analyses revealed that serum CCL18 level was closely linked with primary tumor site, tumor classification, clinical stage, lymph node metastasis and recurrence in patients with LSCC. In contrast, age, gender and histological grade did not correlate to the serum CCL18 level. More importantly, high serum CCL18 level was able to predict the 5-year overall survival time, which was an independent factor associated with the overall survival in patients with LSCC. Therefore, CCL18 is a potentially valuable serum biomarker for the progression and prognosis in patients with LSCC.

CCL18 may directly or indirectly promote tumor angiogenesis, suppress anti-cancer immune response and reshape TME, leading to malignant progression in diverse solid human cancers. CCL18 is mainly expressed and spontaneously secreted by cancer cells and stoma cells including monocytes, macrophages and immature dendritic cells etc. CCL18 secreted from M2-type TAMs is increased not only in chronic inflammations 24, fibrotic diseases 25, 26 and white adipose tissue dysfunction 27, but also in various types of neoplastic diseases. Song's team determined that serum CCL18 was obviously elevated in patients with breast carcinoma, and high CCL18 in the serum was linked with lymph node metastasis and worse histopathological typing, which was an independent influencing factor in the prognosis of patients with breast cancer 12. Yuan etc reported that mean serum CCL18 levels elevated in epithelial ovarian cancer (EOC), which predicted a worse survival status in patients with EOC 19. Similar results were also observed in pancreatic cancer 18, lung cancer 15. In concert with the above available evidence, our study for the first time confirm the clinical significance of serum CCL18 in patients with LSCC, which highlights that serum CCL18 is a valuable biomarker in diverse human cancers. This founding underlines the importance of less-invasive assay of serum CCL18 in clinical management setting, which is more superior to other cancer biomarkers assayed via immunohistochemistry or qPCR in cancer tissue biopsy or surgery.

Although our study only confirms the clinical significance of serum CCL18 in patients with LSCC, CCL18 is also reported to be an efficient molecular target in TME to repress cancer progression. In one respect, CCL18 in TME binds to its receptors (PITPNM3 12, CCR6 28 and CCR8 29-31) on the membrane of tumor cells, activates multiple carcinogenesis associated signaling pathways including NF-κB, PI3K/Akt, Wnt/β-catenin and mTOR etc, which in turn accelerates the progression of diverse human cancers including head and neck cancer 13, 14. On the other hand, CCL18 as a secreted cytokine also remodels TME to promote cancer progression. For example, CCL18 represses the anti-cancer immune response by recruiting regulatory T (Treg) cells and induce an immune suppression environment that finally leads to suppress deleterious inflammation and cancer progression 32-35. Therefore, cytokine CCL18 exert its function via influencing both cancer cells and their surrounding TME.

In conclusion, our study reveals that serum CCL18 is increased in patients with LSCC, which is also linked with diverse malignant phenotypes of LSCC including advanced clinical stages, lymph node metastasis etc. More importantly, examination of serum CCL18 provides useful prognostic information for patients with LSCC. However, how CCL18 participates in the malignant progression of LSCC requires further verification both *in vitro* and *in vivo*.

## Figures and Tables

**Table 1:** The relationship between serum CCL18 and clinicopathological variables in patients with LSCC.

Parameters	Number	Serum CCL18 levels			*P*-value
		Q25	Q50	Q75	
Age					
>56	72	24640.85	34053.28	47889.54	0.425
<=56	74	26698.96	37512.85	54826.48	
Gender					
Female	7	37298.59	46965.44	58073.71	0.116
Male	139	25589.64	34880.33	49652.10	
Primary tumor site					
Glottic	93	22018.72	32587.97	44815.06	**<0.01**
Others	53	31658.10	40500.12	73557.88	
Lymph node status					
N0	94	22158.37	30799.68	38760.60	**<0.01**
N+	52	36791.40	51178.05	89003.81	
T classification					
T1+T2	72	21840.36	29029.14	38884.02	**<0.01**
T3+T4	74	32077.89	40941.73	61300.08	
Clinical stage					
I+II	57	20369.22	26594.64	34894.13	**<0.01**
III+ IV	89	32424.43	42258.68	69677.39	
Histological grade					
G1	15	26333.71	29323.18	49652.10	0.404
G2+G3	131	25861.68	37216.18	52704.01	

**Table 2:** Univariate and multivariate Cox analysis of variables considered for overall survival in patients with LSCC.

Parameters	Overall Survival	*P*-value
	Relative risk (95% CI)	
Univariate		
Gender	2.290(0.315-16.661)	0.413
Age	1.487(0.802-2.755)	0.208
Primary tumor site	2.071(1.122-3.823)	0.020
T classification	2.626(1.339-5.151)	0.005
Lymph node status	2.987(1.601-5.572)	0.001
Clinical stage	5.674(2.224-14.476)	<0.01
Histological grade	0.707(0.297-1.681)	0.432
Serum CCL18	3.212(2.091-4.732)	<0.01
Multivariate		
Serum CCL18	1.538(0.926-3.017)	0.001
Clinical stage	3.929(1.477-10.452)	0.006
